# The Lipocalin2 Gene is Regulated in Mammary Epithelial Cells by NFκB and C/EBP In Response to Mycoplasma

**DOI:** 10.1038/s41598-020-63393-x

**Published:** 2020-05-06

**Authors:** Wei Zhao, Lee Bendickson, Marit Nilsen-Hamilton

**Affiliations:** 1 Roy J Carver Department of Biochemistry, Biophysics and Molecular Biology and the Interdepartmental Molecular, Cellular and Developmental Biology Program, Iowa State University, Ames, IA 50011 USA; 2 Interdepartmental Molecular, Cellular and Developmental Biology Program, Iowa State University, Ames, IA 50011 USA; 3 Present Address: Bayview Physicians Group, Battlefield Medical association, 675 North Battlefield Boulevard, Chesapeake, VA 23320 USA

**Keywords:** Stress signalling, Parasite host response

## Abstract

Lcn2 gene expression increases in response to cell stress signals, particularly in cells involved in the innate immune response. Human Lcn2 (NGAL) is increased in the blood and tissues in response to many stressors including microbial infection and in response to LPS in myeloid and epithelial cells. Here we extend the microbial activators of Lcn2 to mycoplasma and describe studies in which the mechanism of Lcn2 gene regulation by MALP-2 and mycoplasma infection was investigated in mouse mammary epithelial cells. As for the LPS response of myeloid cells, Lcn2 expression in epithelial cells is preceded by increased TNFα, IL-6 and IκBζ expression and selective reduction of IκBζ reduces Lcn2 promoter activity. Lcn2 promoter activation remains elevated well beyond the period of exposure to MALP-2 and is persistently elevated in mycoplasma infected cells. Activation of either the human or the mouse Lcn2 promoter requires both NFκB and C/EBP for activation. Thus, Lcn2 is strongly and enduringly activated by mycoplasma components that stimulate the innate immune response with the same basic regulatory mechanism for the human and mouse genes.

## Introduction

Epithelial cells are the first responders to many pathogenic bacteria and stress conditions *in vivo*. The innate immune response, the first line of defense in the inflammatory response, involves activation of the transcription factors NFκB and C/EBP and their regulators such as IκBζ (also called MAIL, “molecule possessing ankyrin-‌repeats induced by lipopolysaccharide” or INAP, “IL-1-inducible nuclear ankyrin-‌repeat protein”)^1–3^. Inflammation aids in the resolution of infection and also promotes tissue repair, partly by way of the proteins secreted during the process. For example, Lcn2 is highly upregulated in the kidney in response to ischemia-reperfusion injury and macrophages are stimulated by sphingosine-1-phosphate to release Lcn2, which can promote tissue repair^4,5^. Lcn2 is produced by the kidney epithelium^6^ and by epithelial cells from other tissues such as the uterus, mammary gland, liver, stomach, small intestine, colon and lung, both during normal physiological changes that provide opportunities for pathogen invasion and in response to pathogens and other stress inducers^4,7–11^. Lcn2 expression is increased in HeLa cells in response to *M. hominis*^12^. However, although there are many examples of Lcn2 response to LPS [Ibid], the response of epithelial cells of normal tissue origin to MALP-2 or mycoplasma to other pathogenic bacterial forms, such as mycoplasma has not to our knowledge been reported.

*Mycoplasma* spp. are intimately involved in diseases that affect humans^13^ and livestock^14^. Various mycoplasma species are associated with and/or cause diseases including pneumonia, mastitis, arthritis, otitis, genital disorders and keratoconjunctivitis. In humans, several mycoplasma species have been linked to cancer^15–28^. The primary contribution of mycoplasma to cancer and other diseases is most likely their inflammatory properties, which are mediated by the interaction of the lipopeptide MALP-2 with the Toll-like receptor, TLR2/6^29–38^. Cultured cells infected with mycoplasma adopt more cancer-like phenotypes that include activated signaling pathways for proliferation, stimulated migration and the epithelial to mesenchymal transition^18,19,21,34,39,40^. Because mycoplasma infections are important in disease and frequently found in cultured cells that have not been adequately monitored, it is important to understand how these organisms regulate the expression of genes such as Lcn2 that have many reported functions in tissue repair and that are routinely used as monitors of disease status^41–45^.

HC11 epithelial cells, derived from the mouse mammary gland, were chosen for these studies as they possess many characteristics of normal differentiated mammary epithelial cells and Lcn2 is highly expressed during lactation and involution of the mammary gland^9,46,47^. Here we show that Lcn2 gene expression is increased by mycoplasma infection and by MALP-2, the mycoplasma lipopeptide. Activation of the primary response genes NFκB, C/EBP, and IκBζ precedes Lcn2 activation and the Lcn2 mRNA continues to increase for at least 72 h after addition of MALP-2 for which the continued presence of MALP-2 is required. The presence of IκBζ is required for Lcn2 activation by MALP-2. The mouse and human Lcn2 promoters contain proximal NFκB and the C/EBP regulatory elements and the deletion of either element eliminates promoter activation by MALP-2. Thus, Lcn2 responds to inflammatory signals from pathogenic bacteria and mycoplasma by a mechanism that requires IκBζ and involves the direct cooperation of NFκB and C/EBP on the Lcn2 promoter.

## Results

### Gene expression induced by MALP-2

**Figure 1:**
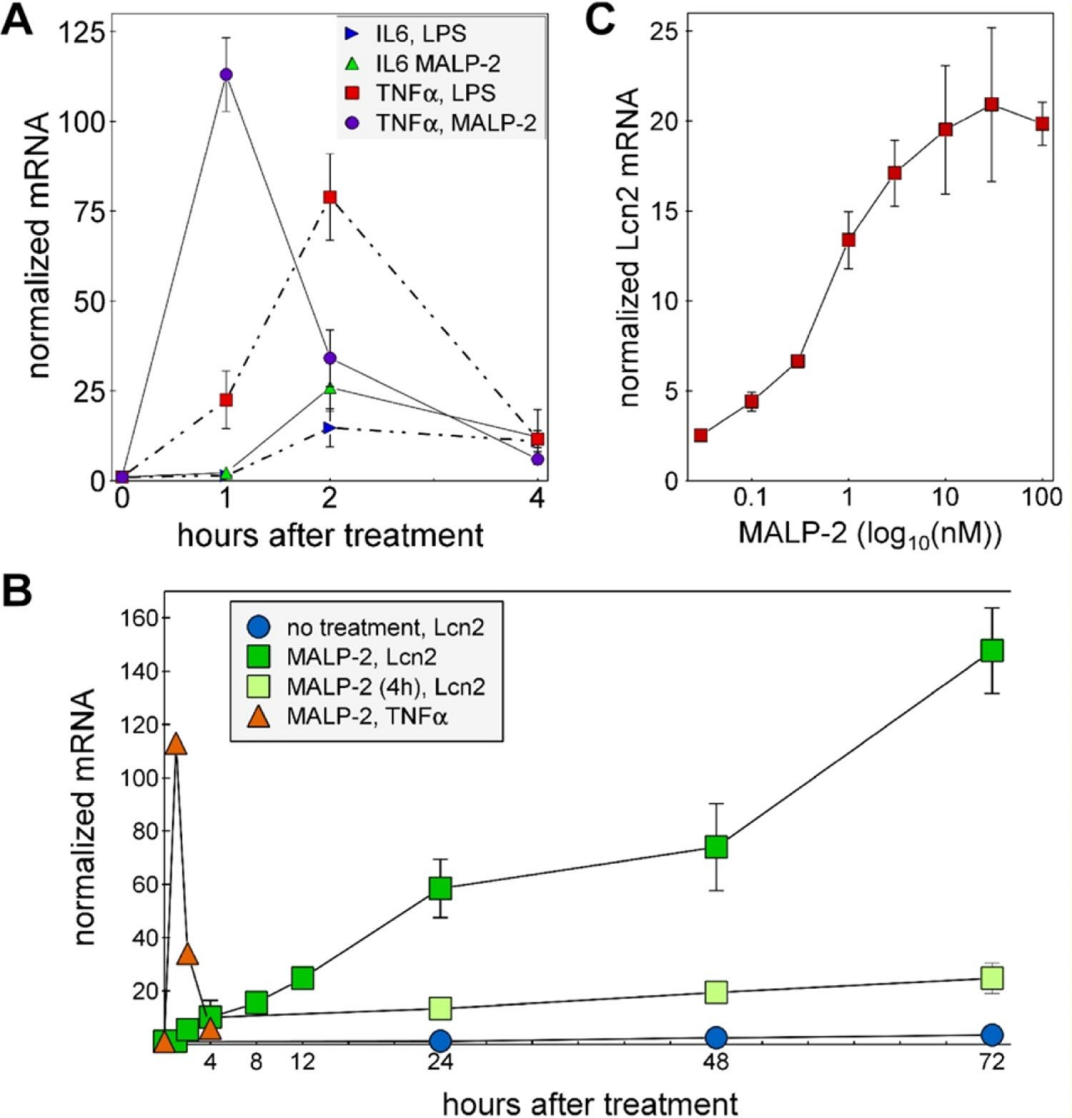
Stress-responsive genes and a persistent increase in Lcn2 expression induced by MALP-2. (**A**) HC11 cells were treated with LPS (10 µg/ml) or MALP-2 (10 ng/ml) for the indicated time periods and RNA samples prepared and quantified for IL-6, TNFα and cyclophilin mRNAs by RT-qPCR. The values for IL-6, TNFα were normalized to the cyclophilin values for the same sample and the zero time point for each gene set was used to normalize the results for all time points of that set. (**B**) HC11 cells were treated with 10 ng/ml MALP-2 for the time periods shown (MALP-2, Lcn2) or treated with MALP-2 for 4 hours then the cells were washed and the medium changed to the same medium lacking MALP-2 [MALP-2 (4 h), Lcn2]. Lcn2, TNFα and cyclophilin mRNA levels were quantified by RT-qPCR and normalized to cyclophilin. (**C**) HC11 cells were treated with MALP-2 at various concentrations for 12 h. Lcn2 and cyclophilin mRNAs were then quantified by RT-qPCR. All values were normalized to the average control value from cells treated with vehicle.

MALP-2 increases the expression of IL-6 and TNFα in HC11 cells, with TNFα gene expression responding to MALP-2 with a peak about one hour earlier than IL6 expression (Fig. 1A). The responses to MALP-2 of these two genes were of a similar elevation as observed after the addition of LPS except that the increase in TNFα mRNA peaked ~1 h sooner in response to MALP-2 than in response to LPS (Fig. 1A) The response of Lcn2 was slower than for IL-6 and TNFα and unlike for these latter genes, Lcn2 expression continued to increase over the course of at least 72 h but only when MALP-2 was present throughout the experiment. When MALP-2 was removed after 4 h, Lcn2 gene expression remained elevated over the remaining 68 h of the experiment (Fig. 1B). The half-maximal response of Lcn2 expression to MALP-2 in HC11 cells, determined using a nonlinear fit model from an average of four independent experiments, was 320 pM (R^2^ = 0.96; Fig. 1C). The increased Lcn2 mRNA levels were accompanied by appearance of Lcn2 in the medium of cells treated with MALP-2 or LPS (Fig. S1) These results show that MALP-2 is a potent inducer of inflammatory response genes and that Lcn2 expression continues to increase for many hours in the presence of MALP-2 and was persistently elevated even after the removal of MALP-2. In contrast, the increased expression of the two cytokines, IL6 and TNFα was rapidly quenched even in the continued presence of MALP-2.

### Elevated expression of inflammatory response genes in mycoplasma infected cells

**Figure 2:**
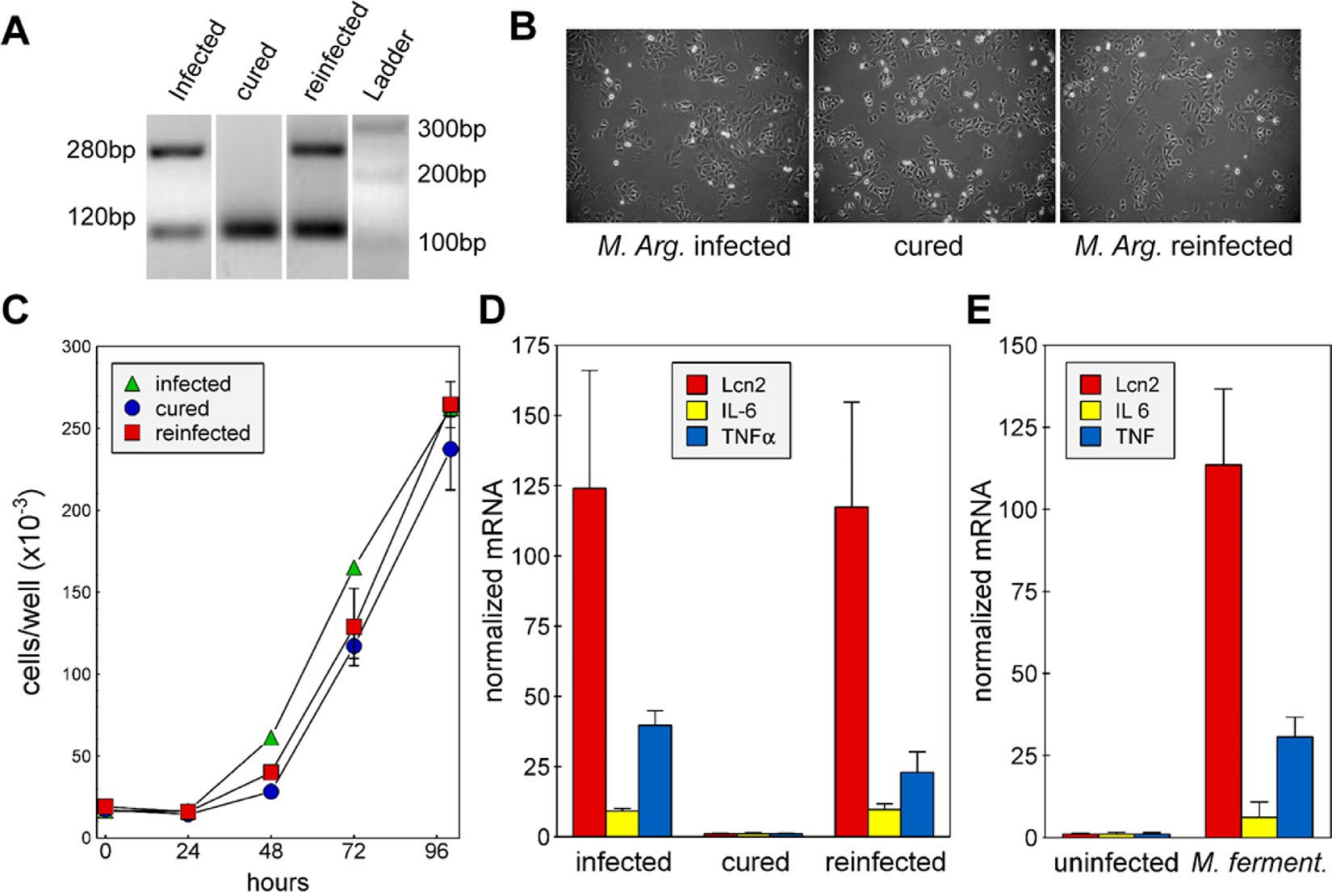
Effect of mycoplasma infection on HC11 cell growth, morphology and gene expression levels. (**A**) Genomic DNAs were amplified with primers targeting the 16 S rDNA of mycoplasma. PCR products were separated by electrophoresis through 2% agarose. The image shows that mycoplasma DNA was amplified from *M. arginini*-infected and reinfected cells, but not from the cured, mycoplasma-free cells. The image is compiled from two gels, which are shown in their entirety the Supplemental materials, (**B**) HC11 cells that were infected or reinfected with *M. arginini*, or uninfected (cured) were seeded on 10 cm plates. Pictures were taken 48 h after seeding. (**C**) HC11 cells, seeded in 24 well plates, were counted after different time periods using a coulter counter. Shown are the means ± standard deviations (SD) from duplicate wells at each time point from a representative experiment. (**D,E**) The expression levels of Lcn2, IL-6 and TNFα and cyclophilin in HC11 cells were quantified by RT-qPCR. The relative mRNA levels in all samples were normalized to those in uninfected (cured) cells. *M. arg*: *M. arginini*-infected cells cured: mycoplasma-free cells, reinfected: cells reinfected with *M. arginini*. *M. ferment: M. fermentans* infected cells.

The identification of a naturally mycoplasma-‌infected HC11 cell culture afforded the opportunity of investigating the effect of chronic mycoplasma contamination on epithelial cells and on the expression of Lcn2. The mycoplasma was identified as *M. arginini* by the sequence of its 16 S rDNA and the cells were freed of *M. arginini* with a BM-cyclin regime. A newly infected cell subline was created using filtered conditioned medium from the parent infected cells. The infection status of each cell subline was verified by PCR (Fig. 2A). All cell sublines exhibited similar epithelial-‌type morphology and growth rates (Fig. 2B,C).

Expression levels of TNFα, IL-6 and Lcn2, determined by RT-qPCR, were higher in the two cell sublines infected with *M. arginini* than in the cured, uninfected cells (Fig. 2D). The elevation of Lcn2 expression in chronically infected cells is consistent with the observed persistent increase in Lcn2 gene expression and the remaining elevation of IL6 and TNFα after 4 h in response to MALP-2 (Fig. 1). We also tested the effect of *Mycoplasma fermentans*, which is one of the 5 species that accounts for more than 90% of cell culture contaminations. HC11 cells infected with *M. fermentans* showed similar increases in the mRNAs of Lcn2, TNFα, and IL-6 as HC11 cells contaminated with *M. arginini* compared with uninfected cells (Fig. 2E).

### NFκB and C/EBP response elements are necessary for M. arginini and MALP-2 induced mouse and human Lcn2 gene expression

**Figure 3:**
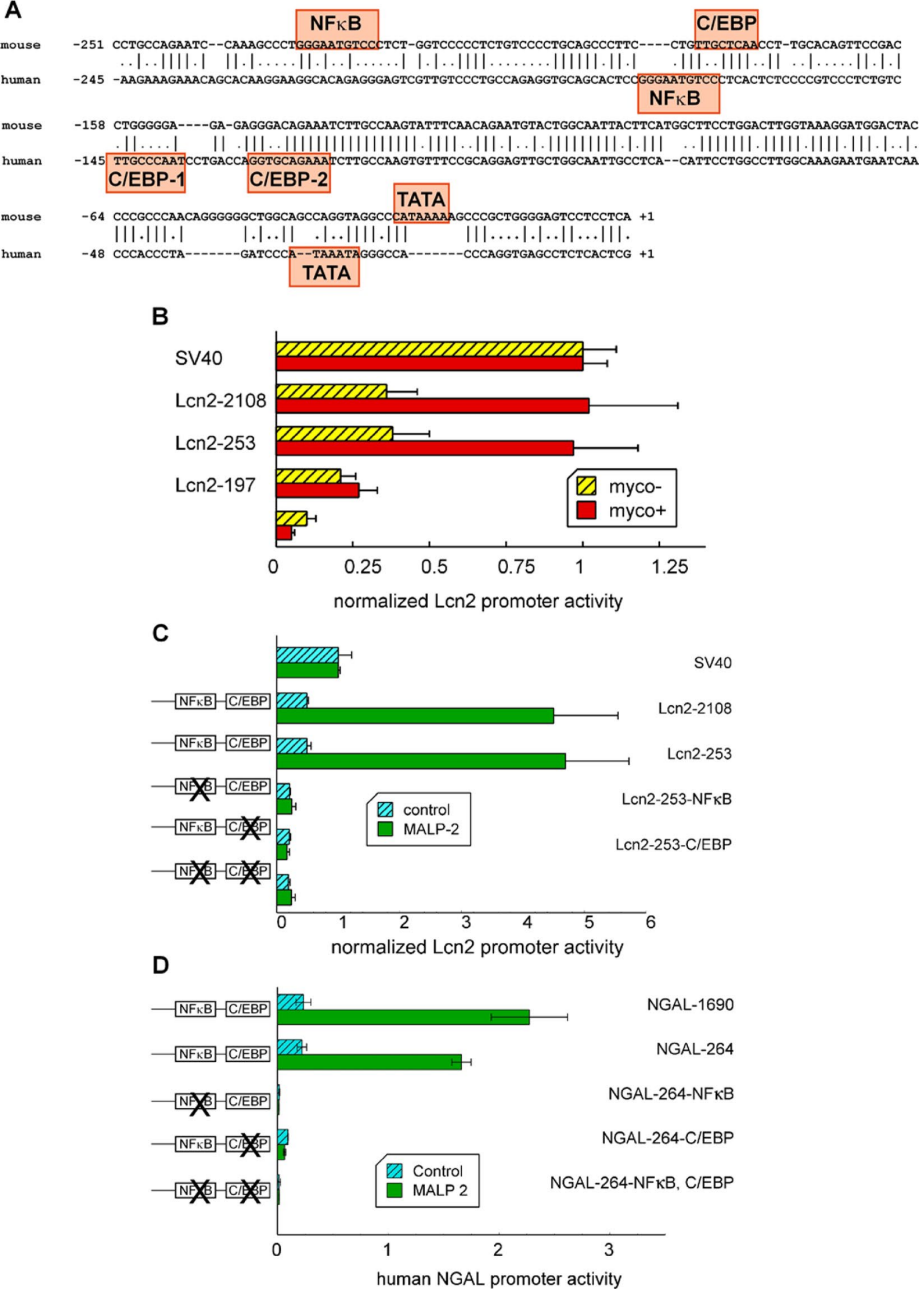
NFκB and C/EBP binding sites are required for MALP-2-induced Lcn2 expression from both the mouse and human promoters. (**A**) An alignment of human and mouse proximal promoters with the C/EBP and NFkB site identified. Human C/EBP-1 was mutated in this study. Sources of the sequences were GenBank: x81627 (mouse) and x99133 (human). (**B**) HC11 cells infected with mycoplasma (myco+) or uninfected (myco-) were co-transfected with a Renilla luciferase expression control plasmid and firefly luciferase reporter plasmids each containing one of a series of truncated Lcn2 promoters (starting at the listed gene position and ending at +53 bp). The cells were harvested and measured for luciferase activity 48 h after transfection. The relative luciferase activities are shown normalized to the value obtained from cells transfected by a plasmid from which firefly luciferase expression was driven by the SV40 promoter. Lcn2-1477, Lcn2-1003, Lcn2-711 and Lcn2-438 had similar activity levels as Lcn2-2108 in *M. arginini* infected HC11 cells (data not shown). (**C,D**) HC11 cells were co-transfected with a Renilla luciferase expression control plasmid and a firefly reporter plasmid with one of the indicated Lcn2 or NGAL promoter segments. Twenty-four hours after transfection the cells were treated with or without 10 ng/ml MALP-2, harvested 12 h later and measured for luciferase activity. Data was normalized to the Renilla transfection control. (**B**) Expression from the mouse Lcn2 promoter and mutant versions. (**C**) Expression from the NGAL promoter and mutant versions. (**A**–**C**) hatched bars, controls; red bars, *M. arginini* infected cells, green bars, MALP-2 treated uninfected cells.

To investigate the mechanism by which MALP-2 and *M. arginini* regulate Lcn2 expression we used mouse and human Lcn2 promoters driving the expression of luciferase as a reporter. A comparison of the activities of a range of truncated mouse Lcn2 promoters showed that the shortest length of promoter to respond to mycoplasma infection was a 253 bp fragment containing the NFκB and C/EBP response elements (Fig. 3A). This is consistent with previous observations that NFκB and C/EBP elements are important for Lcn2 induction by IL-1β and LPS^48,49^.

To test if one or both NFκB and C/EBP elements are required for promoter responsiveness to MALP-2 and *M. arginini* infection, reporter plasmids were constructed of the mouse and human promoters in which one or both sites were mutated to sequences known not to be bound by the respective transcription factor. These reporter genes were transfected into mycoplasma-free and *M. arginini*-infected cells. The mycoplasma-‌free cells were also treated with MALP-2 to determine the requirement of one or both elements for stimulating Lcn2 promoter activity. The results showed that both NFκB and C/EBP elements are required for MALP-2 or *M. arginini* activation of both the mouse and human Lcn2 promoters (Fig. 3B,C).

We investigated which C/EBP isoforms are expressed by HC11 cells by PCR and found evidence for the presence of C/EBPα, β and δ isoforms (Fig. S2). This observation is consistent with the reported isoforms expressed by their mammary gland origin^50^. We examined the effect of MALP-2 on the levels of each C/EBP isoform that was expressed and found no statistically significant increase in ΔΔCt (Student’s Ttest) for their levels of expression with and without MALP-2 incubation for 24 h. C/EBP is present in the nuclei of HC11 cells as expected of this transcription factor (Fig S2). However, unlike for some cells, NFκB was was not found to move extensively into the nucleus after treatment with MALP-2 or LPS. Instead a low level of NFκB was seen in the nucleus at all times with either treatment (Fig. S3).

### IκBζ is a Downstream Regulator from MALP-2 of Lcn2 Expression

**Figure 4:**
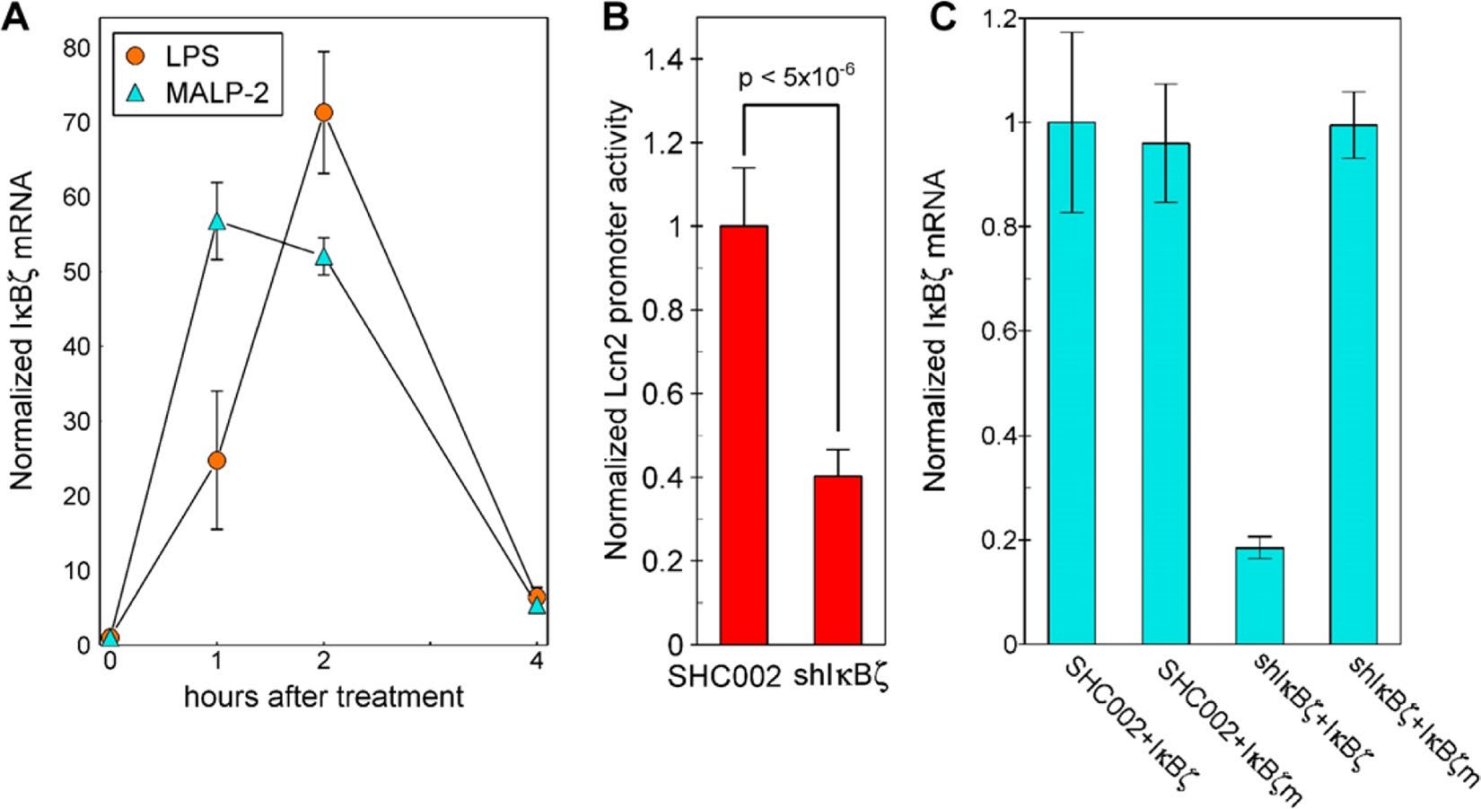
IκBζ is induced by MALP-2 and regulates Lcn2 promoter activity. (**A**) HC11 cells were treated with 10 µg/ml LPS or 10 ng/ml MALP-2. IκBζ mRNA was quantified by RT-qPCR and normalized to the cyclophilin mRNA in the same sample and then all values were normalized to the 0-time point. (**B**) HC11 cells were cotransfected with the 253 bp Lcn2-luciferase plasmid and the Renilla-luciferase control plasmid in combination with a plasmid to express one of the following RNAs: SHC002 or shIκBζ. The transfected cells were treated with 10 ng/ml MALP-2 for 18 h then lysates tested for promoter activity. (**C**) HC11 cells were cotransfected with expression vectors for eGFP with the following combinations: shIκBζ or the SHC002, each in combination with IκBζ or mIκBζ. Samples were collected 24 h after transfection and exogenous IκBζ mRNA was quantified by RT-qPCR with primers that did not amplify the endogenous IκBζ. Each value was normalized to the level of eGFP mRNA in that sample.

IκBζ is a nuclear member of the IκB family that cooperates with NFκB in activating gene expression in monocytes and was demonstrated necessary for human Lcn2 promoter activation in human A549 cells by the combination of IL-17 and TNFα^51^. IκBζ mRNA was tracked with time after addition of MALP-2 or LPS (Fig. 4A). As for the response of TNFα, the increase in IκBζ mRNA peaked about 1 h sooner after the addition of MALP-2 than after LPS. These parallel time courses in response to both MALP-2 and LPS suggest a possible common mechanism of activation of these genes by both compounds.

To test if IκBζ is important for the activation by MALP-2 of Lcn2, the HC11 cells were transfected with short hairpin RNA (shIκBζ). Lcn2 promoter activity was decreased by 60% in HC11 cells stimulated by MALP-2 that were transfected with the 253 bp Lcn2 promoter and cotransfected with IκBζ shRNA compared with those contransfected with the control SCH002 RNA (Fig. 4B). The specificity of shIκBζ in suppressing IκBζ mRNA expression was established using, as a control, an IκBζm mRNA expression vector in which a silent mutation replaced the wildtype sequence in the region complementary to the shIκBζ RNA. This mutant mRNA was not decreased by the transfected shIκBζ mRNA (Fig. 4C).

## Discussion

Lcn2 is an acute phase protein^8^ and its gene expression is increased in response to inflammatory signals in several epithelial tissues including the kidney, stomach, small intestines and lungs^4,7–11^. Increased tissue NGAL (human Lcn2) has been correlated with several diseases that involve stress and inflammation^52^, including kidney disease for which there is a well-studied mouse model^53^. NGAL is also reported as a precocious marker for therapeutic response of renal and non-renal diseases^54^. As a result of these and many other studies, NGAL has been adopted as a blood marker for tissue inflammation and an indicator of kidney injury^42,55–59^.

In cell culture Lcn2 is induced by growth factors^60,61^, cytokines^7,62–64^, LPS^11,65,66^, glucocorticoids^9,66,67^, and MALP-2 (this work). The half maximal response to MALP-2 of 320 pM is in the range of the half maximal responses of 30 to 70 pM for *E. coli* LPS^68–70^, and 1.3 nM for *S. Minnesota* LPS^70^. This result is also consistent with a previous study showing that MALP-2 is a potent macrophage stimulatory lipopeptide that increases NO release from macrophages at concentrations in the picomolar range^71^. Lcn2 is expressed by epithelial cells, which coordinate with macrophages and dendritic cells to mount the innate immune response. These data show that epithelial cells are stimulated by the same concentration range of MALP-2 that activates macrophages. Thus, it is expected that both epithelial cells and macrophages are activated by MALP-2 *in vivo* as a result of mycoplasma infection.

Lcn2 gene expression is activated in cells that mount the innate immune response including epithelial cells, dendritic cells, macrophages and neutrophils^11,66,72^. Mouse Lcn2 has been demonstrated to suppress bacterial proliferation by complexing iron siderophores released by pathogens in an *in vivo* model^73^. Studies of rodent models have demonstrated that Lcn2 promotes tissue repair in the stomach^4^, kidney^5,74^, skeletal muscle^75^, and small intestine^76^, Several effects of the Lcn2 protein in cell culture may be linked to these *in vivo* observations, including effects of Lcn2 on cell proliferation and movement^4,5,77^. By contrast, Lcn2 is also associated with apoptotic defects in hematopoietic cells^78,79^, promoting the epithelial mesenchymal transition^80–82^ and opposing cutaneous wound healing in diabetic mice^83^. Thus, the role of Lcn2 *in vivo* is likely to be complex and even cell type-dependent.

We examined the response of HC11 mammary-derived epithelial cells to mycoplasma and its MALP-2 product. As an externally exposed tissue, the mammary gland is susceptible to bacterial infection, which results in mastitis, an inflammatory condition that has been studied extensively in agricultural animals because of its large economic impact. Infections by pathogenic mycoplasma also induce persistent host inflammation^84–87^, which is consistent with the many reports of the association of mycoplasma infections and cancer^15–28^. In response to bacterial infection, epithelial cells, tissue-resident macrophages and dendritic cells (DCs) respond due to recognition of the PAMPs (pathogen associated molecular patterns) by pattern recognition receptors such as the Toll-like receptors (TLR)^88–90^. The *M. fermentans*-derived lipopeptide, MALP-2, with the sequence (S-[2,3-bisacyl(C16:0/C18:0;‌C18:1)-‌oxypropyl]‌cysteine-‌GNNDE‌S‌‌NISFKEK, signals through TLR2 and TLR6^71,91,92^. Activation of TLRs results in the release of proinflammatory cytokines and other inflammatory mediators^93,94^. Here we demonstrate a rapid increase in cytokine mRNA with the relatively faster increase in TNFα compared with IL6 being consistent with previous reports of the relative time courses of increased secreted TNFα and IL6 in response to MALP-2^95,96^.

Our results show that *M. arginini, M. fermentans* and MALP-2 each stimulate the expression of TNFα, IL6 and Lcn2 expression in HC11 epithelial cells. TNFα and IL-6 are regulated by C/EBP and NFκB as we show here for Lcn2. As for their mammary gland origin^50^, HC11 cells express several C/EBP isoforms. MALP-2 does not increase the expression of any isoform and we have located C/EBPβ in the nucleus where it and the other C/EBP isoforms are expected to be found. The sequence preferences of the C/EBP isoforms for their binding elements are similar and the distinction of which isoform functions in a particular cell or tissue is determined by the isoform expression pattern^97^. Thus, more than one C/EBP is likely to be involved in regulating Lcn2 gene expression in HC11 cells. We also note that the previous literature is controversial with respect to C/EBP involvement in regulating Lcn2. But, all previous studies of which we are aware have been done in cells lines of embryonic origin^48,98^ or tumor origin^48,51,99^. Ours is the only study to our knowledge of the regulation of Lcn2 expression in a cell line originating from a normal epithelial source.

Inflammatory stimuli can stimulate translocation of NFκB from the cytoplasm into the nucleus. Although both activators greatly increased the expression of Lcn2 by HC11 cells, neither caused a large change in the distribution of NFκB. This observation is consistent with results already reported for untransformed epithelial cells. In H4-1 non-transformed human neonatal small intestinal cells only 10% or few (depending on the conditions) of these cells demonstrate NFκB translocation in response to a bacterial inflammatory stimulus^100^. By contrast, NFκB was translocated into the nucleus of a majority of A549 cells^101^ and HeLa cells^102^. Thus, the translocation response of NFκB is context dependent and may be a more prominent feature of transformed cells than cells that display a normal phenotype.

In contrast to the rapid quenching of the TNFα and IL6 expression after activation by MALP-2, Lcn2 expression continued to increase over 72 h. Our data suggests that the extended period of response of Lcn2 to MALP-2 is driven by the early increase in IκBζ expression, which activates secondary response genes but not primary response genes in macrophages and macrophage cell lines by a process that involves histone H3K4 trimethylation and results a persistent elevation in gene expression^103^. Epigenetic modification as a mechanism by which IκBζ regulates Lcn2 expression is consistent with the observation that Lcn2 remained elevated for many hours after the removal of MALP-2.

The expression IL6 and TNFα was found to be constitutively elevated in cells chronically infected with mycoplasma, which is consistent with the observed persistence of the mammary inflammatory response to mycoplasma infections^104^ and suggests possible epigenetic changes in these genes in response to mycoplasma infection.

In summary, we have shown that mycoplasma and the mycoplasma-derived membrane lipopeptide, MALP-2, induce the expression of inflammatory response genes in epithelial cells. As well as contributing to chronic inflammation *in vivo*, the elevated expression of Lcn2 and cytokine genes in mycoplasma-infected cells reflects a inflammatory condition involving a subset of genes that alters the proteins expressed by the infected cells^105^, which can impact the outcomes of experiments performed with such cells in undefined ways and thus influence interpretation of the experimental results. The mechanism of Lcn2 induction in mammary epithelial cells by MALP-2 is similar to that in myeloid cells induced by LPS with the involvement of NFκB, C/EBP and IκBζ^30,35,106^. Compared with the brief period of increase in the cytokine genes in response to MALP-2, the Lcn2 response continues for at least 72 h and the elevated expression persists for long after the removal of MALP-2. We show by mutation and molecular manipulation that the presence of IκBζ and the proximal NFκB and C/EBP transcription elements in the Lcn2 promoter are required for its activation.

## Materials and Methods

### Materials

LPS was from Sigma-Aldrich (St. Louis, MO) and MALP-2 was from Axxora (San Diego, CA). The IκBζ shRNA and SCH002 control plasmids were from Sigma (St. Louis, MO), the eGFP expression plasmid was from Clontech (Mountain View, CA) and pcDNA3.1 was from Invitrogen (Carlsbad, CA).

### Cell culture

HC11 cells, a clone from the COMMA-D mouse mammary gland cell line^47,107^, were obtained from Dr. L. Sheffield (University of Wisconsin, Madison, Wisconsin) with permission of the cell line’s originator, Dr. B. Groner Institute for Experimental Cancer, Freiburg, Germany,^47^ and cultured at 37 °C in RPMI 1640 media (Sigma, St. Louis, MO) with 2% FCS (Hyclone, Logan, UT), 5 μg/ml insulin (Sigma, St. Louis, MO), 10 units/ml each of penicillin and streptomycin with 5% CO_2_. Experiment treatments were in the same medium but with 0.1% FCS. HC11 cells were seeded in 6-well plates for 24 h in 2% FCS culture medium and then switched to 0.1% FCS culture medium. Sixteen hours later, MALP-2, LPS or the vehicle control were added to the medium. Cells were harvested at various times for RNA or protein analysis.

### Mycoplasma detection, removal and infection

Genomic DNA, prepared using DNAzol (Invitrogen), was used as the template in PCR to detect mycoplasma. The primers in this reaction corresponded to a region of the 16 S rDNA that is highly conserved in mycoplasma^108^. The primer sequences were: sense ACCATGCACCAYCTGTCAYTC and anti-‌sense GAGCAAACAGGATTAGATAC. An internal reference control was included with every sample from which a band of 120 bp was amplified. The samples were amplified for 28 cycles of 94 °C for 30 sec, 55 °C for 60 sec, then 72 ^o^C for 60 sec. A band of 280 bp indicates mycoplasma contamination^108^. This band and another region of the mycoplasma 16 S rDNA were sequenced from an infected cell culture and the mycoplasmal contaminant was identified as *M. arginini*.

Infected cell cultures were cleaned of *M. arginini* by treating the cells with three cycles of 10 µg/ml BM-cyclin 1 (Roche Applied Science, Indianapolis, IN) for 3 days followed by 5 µg/ml BM-cyclin 2 (Roche) for 4 days. The cells were reinfected with *M. arginini* by exposing them to conditioned medium collected from an *M. arginini* infected cell culture that had been filtered through a 0.22 µm sterilized filter. Mycoplasma-free HC11 cells were also infected with *M. fermentans* (ATCC, Manassas, VA). The infected cells were transferred for three passages before the mycoplasma could be detected by PCR amplification.

### RNA extraction and reverse transcriptase quantitative PCR (RT-qPCR)

Total RNA was extracted using the Trizol Reagent (Invitrogen, Carlsbad, CA) according to the manufacturer’s instructions. The total RNA were dissolved in DEPC-treated H_2_O, quantified by spectrophotometry and stored at −80 ^o^C until use. To remove contaminating DNA, 1 μg total RNA was incubated for 15 min with 1 unit DNase (Invitrogen) then the DNase was inactivated by exposure to 70 °C for 15 min. Reverse transcription was done with Superscript II (Invitrogen) and an 18 nt oligo dT as primer. qPCR was performed in an Opticon (MJ Research, Waltham, MA) using the FullVelocity™ QPCR Master Mix (Stratagene, La Jolla, CA). Levels of each mRNA gene product was normalized to the cyclophilin level in the same sample. Primers used in the experiments were 1) mouse Lcn2 sense: AATGTCACCTCCATCCTGGTCA, anti-sense: GCGAACTGGTTGTAGTCCGTGGT; 2) cyclophilin sense: CTTTTCGCCGCTTGCTGCA, anti-sense: ACCACCCTGGCACATGAATCCT; 3) IL-6 sense: GAGGATACCACTCCCAACAGACC, anti-sense: AAGTGCATCATCGTTGTTCATACA; 4) TNFα sense: CATCTTCTCAAAATTCGAGTGACAA, anti-sense: TGGGAGTAGACAAGGTACAACCC; 5) IκBζ sense: TGCAGAGGAATCGGCAGTCT, anti-sense: CGGACTGCGTCCAACTGTGT; 6) exogenous IκBζ sense: CACCGCCCTCCATGTTGCT, anti-‌sense: GCAAACAACAGATGGCTGGCA; and 7) eGFP sense: ACAAGCAGAAGAACGGCATC, anti-sense: ACGAACTCCAGCAGGACCAT. For each mRNA analysis a standard curve was used to determine the quantity of the amplified cDNA in each sample. The standard curve was created by amplifying, under the same conditions as the cell-derived samples, a range of known amounts of PCR amplicons (from 10^−6^ to 8 pg/well) using a template with the same sequence as present in the cDNA being quantified. Standard curves were plotted as log(pg/well) vs. C(t). Samples with C(t)’s outside the range of the standard curve were not included in the quantitative data and appropriate dilutions of the cDNA were reanalyzed to obtain quantitative values within the range of the standard curve.

### Cloning

Mouse Lcn2 promoters of various lengths were prepared as PCR products from a mouse genomic BACmid (ES17526, Genomesystems Inc.) template. Fragments were inserted into the luciferase reporter plasmid pGL3-basic (Promega, Madison WI). Primers used for gene truncations were 1) −2108bp, sense: CAGACACAACAGAAGAGGGCAT, 2) −1477bp, sense: TGTGGGTTGTGTGAGGCTGTA, 3) −1003bp, sense: CAGGGCAGTGTGGAGACACA, 4) −711bp, sense: GCAGCCACATCTAAGGACTACG, 5) −438bp sense: GGTCTGGTGTTCAGATGGCTT), 6) −253bp sense TGCCTGCCAGAATCCAAAG, 7) −197bp, sense: CAGCCCTTCCTGTTGCTCA, 8) −108bp, sense: GCAATTACTTCATGGCTTCCTG, 9) −253-NFκB,GGG‌/AAA: sense: TGCCTGCCAGAATCCAAAGCCCTAAAAATG with the same anti-sense oligonucleotide starting at +53 bp used for all: GGTTTCCACAGCTACTAGGTCTGA. Mutagenesis of the promoter was done using the Quickchange II kit (Stratagene) to convert the C/EBP binding site from CAGCCCTTCCTGTTGCTCAACCTTGCACAGTTCCGAC to CAGCCCTTCCTGGCACTTGGCCTTGCACAGTTCCGAC and/‌or the NFκB binding site from GCCCTGGGAATG to GCCCTAAAAATG where the changed bases are underlined.

Human Lcn2 (NGAL) promoters of various lengths were also inserted into pGL3-basic. −1690bp was amplified with sense: AGTCGGTACCGATCTCGGCTCACTG‌CAAC and anti-‌sense: GTCACTCGAGGCAGGCGCTGTGGTG (+51) using pNGP1695CAT (kindly donated by N. Borregaard) as template. −264bp was amplified from human genomic DNA (extracted from human HCT-8 cells) with sense: AGTCGG‌TAC‌CCCAC‌ATA‌CAG‌G‌G‌CAATCAGA and anti-sense: GTCACTCGAGGATTT‌CAGGGCCGAGGAAG (+69). The Quickchange II kit was used to introduce mutations: the NFκB binding site from AGGTGCAGCACTCCGGGAATGTCCCTCACTCT to AGGTGCAGCAC‌TCCAAAA‌ATG‌T‌C‌C‌CTCACTCT; the C/EBP binding site from CCCGTCCCTCTGTCTTGCC‌CAATCC‌TGACC‌A‌G‌G‌TGCAG to CCCGTCCCTCTGTC‌‌GCACCTGGTCCTGACCAGGT‌GCAG with the changed bases underlined.

IκBζ cDNA was amplified from a preparation of HC11 RNA and cloned into pcDNA3.1(-) (Invitrogen). A silent mutation in the region that is complementary to sh IκBζ in the wildtype mRNA was created using the Quickchange II kit (Stratagene) to convert the sequence GCCCTGCTTCAGAATATTATA to GCCCTG‌CTCCAAAACATTATA in which the changed bases are underlined.

### Luciferase assay

To measure the activities of the Lcn2 promoter and its truncations, HC11 cells were co-transfected using Lipofectamine 2000 (Invitrogen), with the appropriate promoter-‌luciferase reporter plasmids in combination with the plasmid pRLSV-40 (Promega) from which the Renilla luciferase is expressed under the control of the SV40 promoter. The activities of the two luciferases were quantified by the Dual Luciferase assay (Promega) using a Glomax 20/20 luminometer (Promega). The Renilla luciferase activity provides an internal control for the cell lysate content of the sample. The measure of promoter activity was calculated by dividing the firefly luciferase activity by the Renilla luciferase activity in each sample.

### Statistical and quantitative analysis

Triplicate independently treated samples were quantified for RT-qPCR or luciferase and the average of the three values were taken as the result for that treatment or condition. All results shown were tested with at least two and up to 7 independently performed experiments in which similar results were obtained. The error bars show the standard deviations of the means.

## Supplementary information

Supplementary Information.

## Data Availability

All data on which this work is based  is available upon request to MNH.
