# Supplementary material for: The Lipocalin2 Gene is Regulated in Mammary Epithelial Cells by NFκB and C/EBP In Response to Mycoplasma
Source: Sci Rep. 2020 May 6;10:7641. doi: 10.1038/s41598-020-63393-x (PMC7203223; doi:10.1038/s41598-020-63393-x)
Supplement: Supplementary file 1 — Supplementary Information. [file 41598_2020_63393_MOESM1_ESM.pdf]

The Lipocalin2 Gene is Regulated in Mammary Epithelial Cells by NFκB and C/EBP In Response to Mycoplasma

Wei Zhao,[1,2,3] Lee Bendickson[1] and Marit Nilsen-Hamilton[1,2]

Supplementary Material

Table S1: Oligonucleotides used for supplemental materials

C/EBP isoform	Amplicon size (nt)	primers
C/EBPα	329	TTCGGGTCGCTGGATCTCTA,<br>CCCGAGAGGAAGCAGGAATC
C/EBPβ	130	GGTTTCGGGACTTGATGCA<br>CAACAACCCCGCAGGAAC
C/EBPγ	399	GCAAAGGAACGTGCCCAAAT<br>GTGCGCATGCTCAAGAAACA
C/EBPδ	189	GAACCCGCGGCCTTCTAC<br>GAAGAGTTCGTCGTGGCACA
C/EBPε	243	AGTCGAGGCAGCTACAATCC<br>GTTACGTTCACGTCGCAGTC

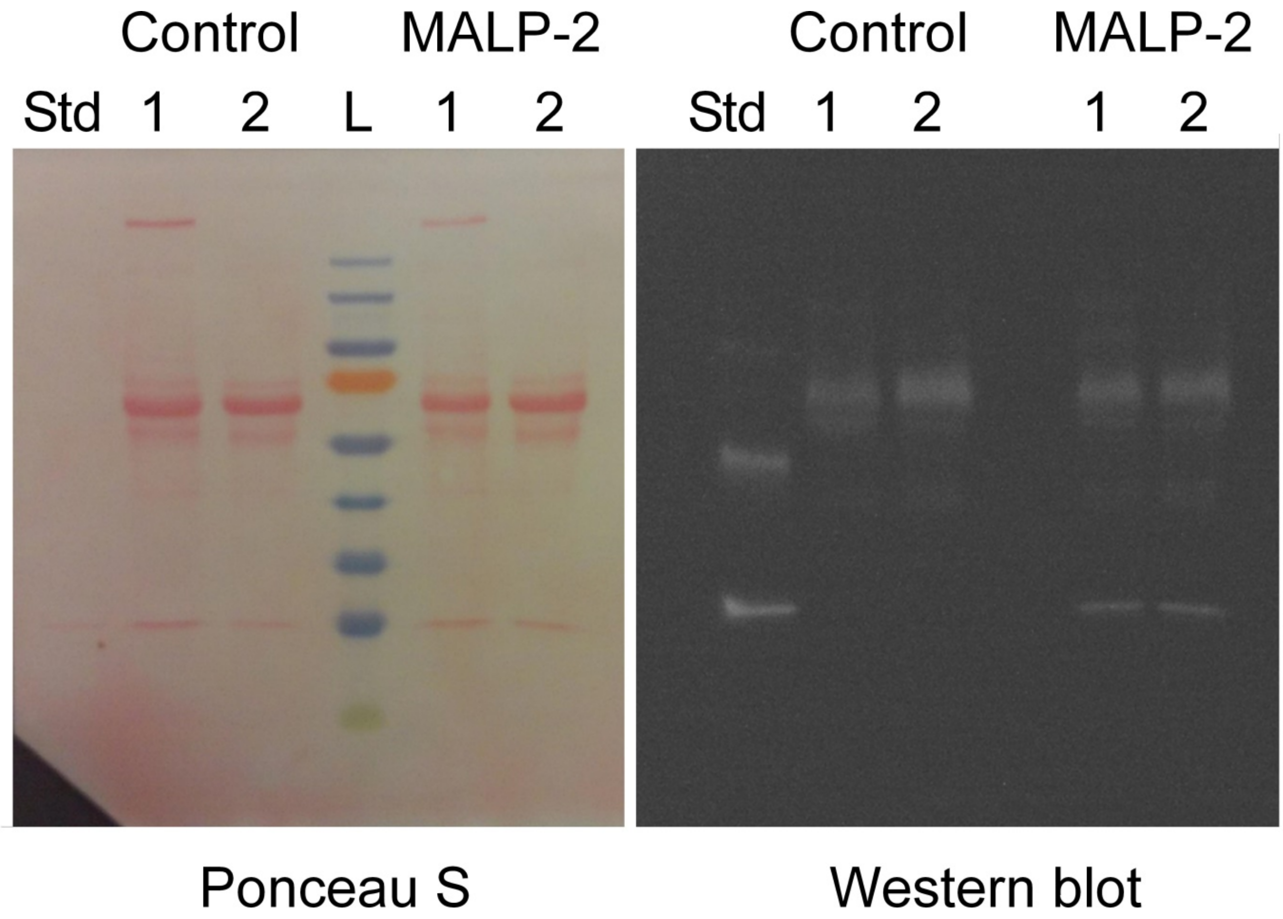

Figure S1: Effect of MALP-2 on secreted levels of Lcn2

Legend: HC11 cells were grown in RPMI-1640, 5% FCS26, 100U/ml P/S, 5ug/ml insulin until 50% confluent then switched to the same medium with 0.1% FCS for 24 h after which they were incubated with or without 10 ng/mL MALP-2 for 24 h. The medium was collected, concentrated and resolved on a 15% SDS acrylamide under reducing conditions. The gel blotted to a nitrocellulose membrane and the membrane immunostained for Lcn2 using primary polyclonal antibody produced against purified murine Lcn2 (1:500) and goat anti-rabbit IgG-HRP from Pierce (catalog#31462) at 1:5,000. The immunostain was developed using chemiluminescent substrates (Pierce catalog#32209). A standard of purified Lcn2 (Std) was run in the first lane of the gel. **Left panel:** Ponceau S stain of the membrane after transfer showing remaining protein and the ladder (L), PageRuler^{Tm} from Thermo Scientific (catalog# 6616). **Right panel:** Immunostained gel. The results of two independent experiments (1 and 2) are shown.

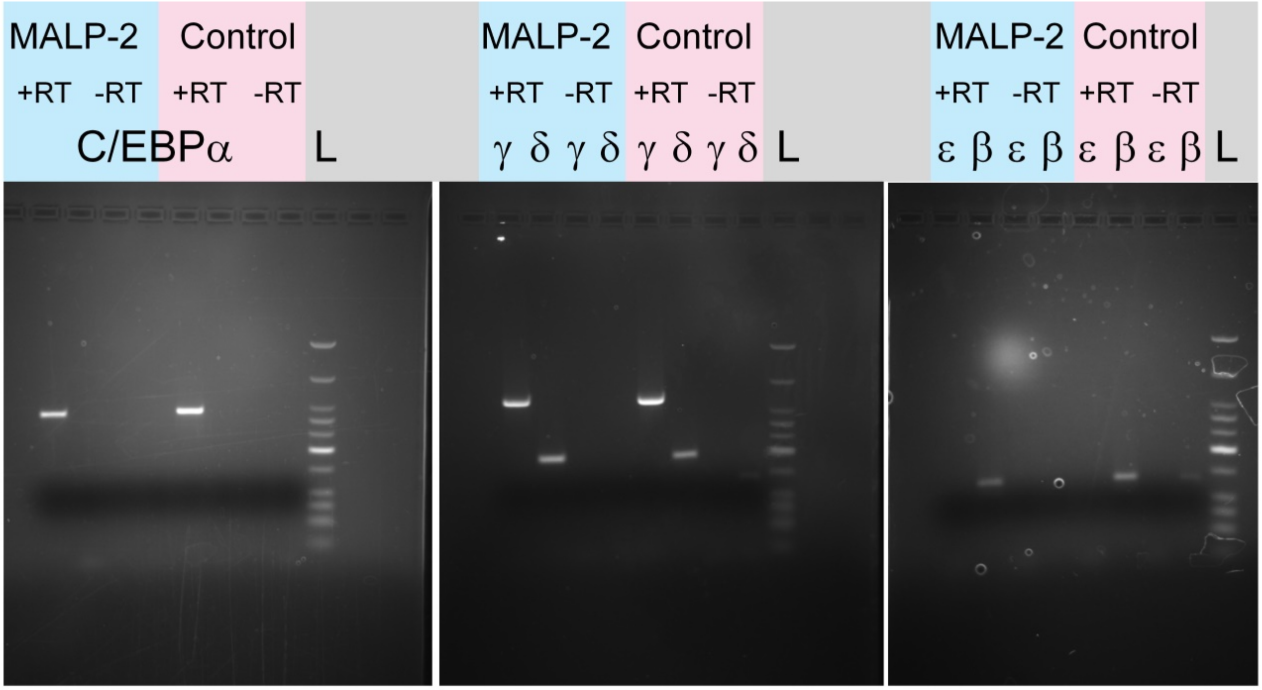

Figure S2: C/EBP isoforms expressed by HC11 cells

Legend: HC11 cells were grown in RPMI-1640, 5% FCS26, 100U/ml P/S, 5ug/ml insulin until 50% confluent then switched to the same medium with 0.1% FCS for 24 h after which they were incubated with or without 10 ng/mL MALP-2 for 24 h. The cells were collected, the RNA extracted with Trizol reagent (Sigma-Aldrich). The RNA was incubated for 15 min at 23ºC with 1 U/µL Dnase (Invitrogen catalog#18068015) then reverse transcribed in the presence (+RT) or absence (-RT) of 10 U/µL SuperScript IV, Thermofisher (catalog# 18090010) for 23, 50 then 80ºC for 10 min at each temperature. The resulting cDNAs were quantified in 50% iQ SYBR Green supermix (BioRad catalog#170-8880) using primers in table S1 to amplify C/EBPα, β, γ, δ, or ε. The samples were evaluated by real-time PCR for estimates of Ct and then resolved by electrophoresis through 2% agarose in TAE buffer. A low molecular weight DNA ladder (New England Biolabs, catalog # N3233S) was run on each gel.

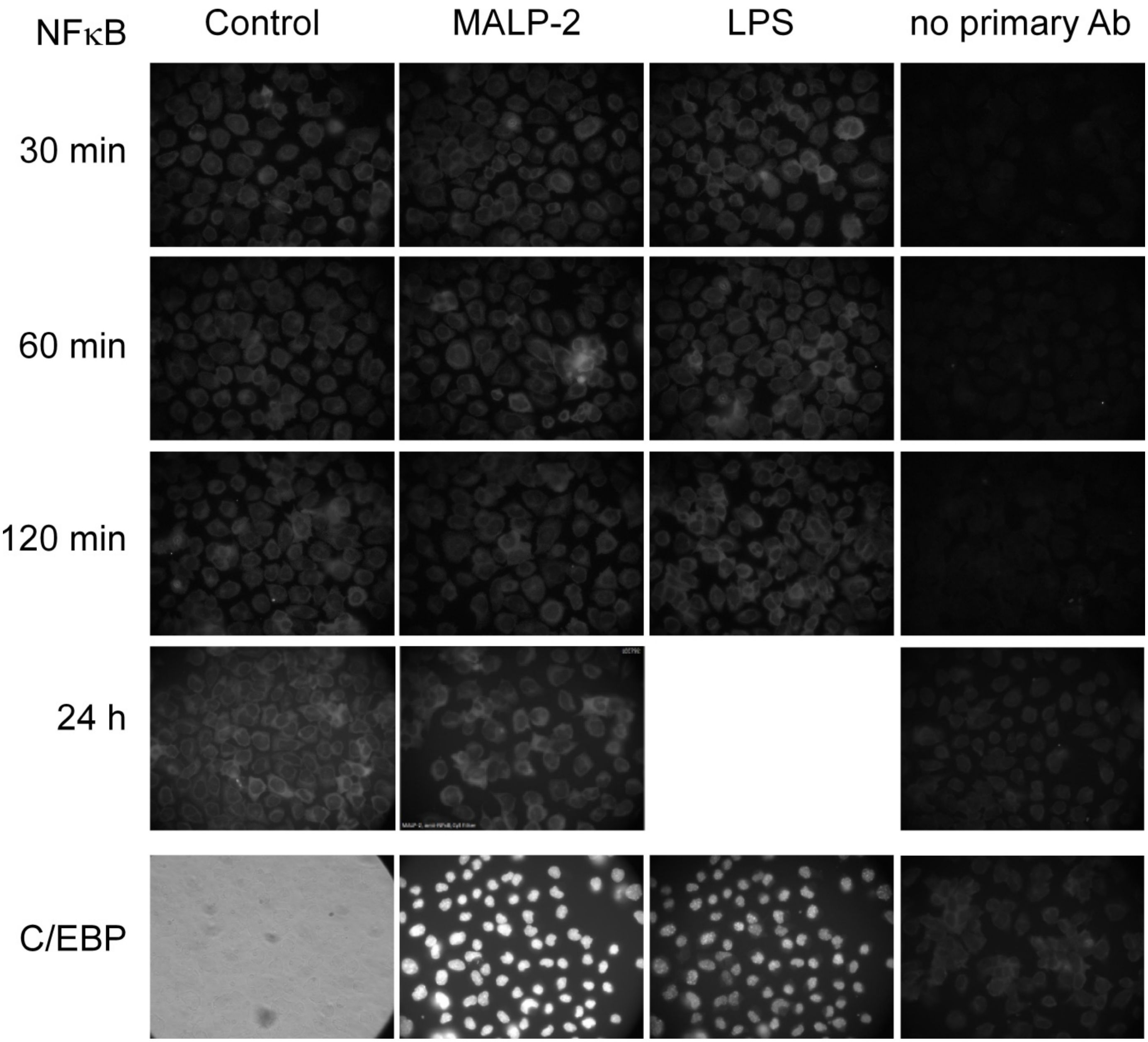

Figure S3: Effect of MALP-2 and LPS on NFκB distribution in HC11 cells

Legend: HC11 cells were grown in RPMI-1640, 5% FCS26, 100U/ml P/S, 5ug/ml insulin until 50% confluent then switched to the same medium with 0.1% FCS for 24 h after which they were incubated with or without 10 ng/mL MALP-2 or 10 µg/mL LPS for 30, 60, or 120 min (experiment 1) or 24 h (experiment 2). The cells were then fixed and immunostained with anti-NfκB p65 (F-6), Santa Cruz Biotechnology, Inc. #sc-8008x (primary antibody) and Cy3 AffiniPure Donkey Anti-Mouse IgG, Jackson ImmunoResearch Laboratories, #715-165-151, lot# 67310. (secondary antibody). The blocking solution was 154 mM NaCl, 3 mM Na_2HPO_4, 1 mM KH_2PO_4, pH 7.4), 0.4% BSA, 0.2% Triton X-100, 2% donkey serum.
